# Nutrient Enrichment Increases Mortality of Mangroves

**DOI:** 10.1371/journal.pone.0005600

**Published:** 2009-05-19

**Authors:** Catherine E. Lovelock, Marilyn C. Ball, Katherine C. Martin, Ilka C. Feller

**Affiliations:** 1 Centre for Marine Studies and School of Biological Science, University of Queensland, St Lucia, Queensland, Australia; 2 Functional Ecology Group, Research School of Biological Sciences, Australian National University, Canberra, Australian Capital Territory, Australia; 3 Smithsonian Environmental Research Center, Edgewater, Maryland, United States of America; Monash University, Australia

## Abstract

Nutrient enrichment of the coastal zone places intense pressure on marine communities. Previous studies have shown that growth of intertidal mangrove forests is accelerated with enhanced nutrient availability. However, nutrient enrichment favours growth of shoots relative to roots, thus enhancing growth rates but increasing vulnerability to environmental stresses that adversely affect plant water relations. Two such stresses are high salinity and low humidity, both of which require greater investment in roots to meet the demands for water by the shoots. Here we present data from a global network of sites that documents enhanced mortality of mangroves with experimental nutrient enrichment at sites where high sediment salinity was coincident with low rainfall and low humidity. Thus the benefits of increased mangrove growth in response to coastal eutrophication is offset by the costs of decreased resilience due to mortality during drought, with mortality increasing with soil water salinity along climatic gradients.

## Introduction

Nutrient enrichment is one of the most serious threats to near shore coastal ecosystems [1], [2]. The consequences of nutrient enrichment include algal blooms [3], coral reef degradation [4], [5], loss of diversity and ecosystem resilience [6], [7] and, in extreme cases, the development of “dead” zones” [8]. These negative consequences contrast with observations that marine plant growth, including that of tropical mangroves is enhanced with nutrient enrichment [3], [9]–[19]. Indeed, for decades mangroves have been proposed as suitable for use in sewage and aquaculture effluent treatment [20]–[22], although assessment of long-term consequences of nutrient enrichment for mangrove ecosystems is lacking.

There is reason to expect the addition of nutrients will have detrimental effects on mangrove forests. As nutrient availability increases, plants invest less in roots and more in aboveground parts, thereby further enhancing growth rates [23]–[26]. However, it follows that plants exposed to high levels of nutrient availability must have greater susceptibility to environmental stressors, such as drought, that require large investment in roots for tolerance [27]. Mangroves characteristically increase allocation of carbon to growth of roots relative to shoots with increase in salinity, with this pattern being amplified with decreasing humidity [28], [29]. These observations invite two hypotheses: 1) that mangroves exposed to high nutrient availability, with their relatively lower investment in roots and greater investment in canopies, should suffer greater mortality during drought, and 2) that nutrient-induced mortality should be particularly high in sites subject to periods of low and fluctuating rainfall and humidity and high sediment salinity.

Here we report on the long term survivorship of mangrove trees that have been experimentally fertilized at 12 sites for 3 to 12 years (Table S1). Our sites ranged over two distinct biogeographic provinces. They were located within the Caribbean basin and in the Indo Pacific region, where they were situated in New Zealand and on the west and east coasts of Australia. Within sites, we fertilized trees in two or more distinct environments and where possible we fertilized multiple species. Trees were fertilized in seaward fringing forests that are inundated frequently (9 experiments) and also in scrub forests that are less frequently inundated and where soils are often hypersaline (16 experiments). The mean annual rainfall ranged over our sites from 0.3 m in Exmouth, Western Australia to >4 m in Bocas del Toro, Panamá. We have previously shown that nutrient enrichment increases growth over our wide range of sites (12, 14–19, Table S1) and here we show that tree mortality is also increased with nutrient enrichment.

## Results

There was a marked difference between tree mortality in seaward fringing forests and landward scrub forests. No unfertilized trees died within the fringe forest where soil water salinities were similar to those of ambient tidal flood water, averaging 37 ppt over all sites. Similarly, no tree death occurred in response to nutrient additions in fringe forests. In contrast, death of unfertilized trees occurred in landward scrub forests where hypersaline soils developed at sites with low annual rainfall (Fig S1). This pattern was accentuated by the addition of nitrogen (N) fertilizer, but not P fertilizer, with tree death occurring in response to N fertilization at sites with lower annual rainfall than other treatments. Time since fertilization began (years of experimental fertilization) did not influence tree mortality. Over all scrub forest sites, tree death was significantly correlated with the mean salinity of soil porewater (Kendall's Tau 0.36, *P* = 0.035) and negatively correlated with mean annual rainfall (Kendall's Tau −0.34, *P* = 0.039). The probability of tree survival was significantly lower in N-fertilized than P-fertilized or unfertilized trees (Fig S2).

A period of particularly low rainfall on the Queensland coast of northeastern Australia provided an opportunity to assess canopy loss over treatments in scrub forests at two sites differing in soil water salinity (Fig S3). Soil water salinity was higher at the Port Douglas site than at the Hinchinbrook Channel site (75 vs 60 ppt). Fertilization with N resulted in greater canopy loss than observed in unfertilized trees or those fertilized with P. Averaged over all treatments, trees at the more saline Port Douglas site suffered greater canopy loss than those at the less saline Hinchinbrook Channel site (35% compared to 22%).

## Discussion

Our results show that increasing nutrient availability introduces an instability into mangrove forests that lowers their resilience to environmental variability. The instability arises because nutrients, particularly N, stimulate growth of shoots relative to roots [30], thereby enhancing productivity during favourable periods but increasing vulnerability to water stress during drought. Such stress can become acute where hypersaline soils limit the capacity for water uptake by roots while low atmospheric humidity exacerbates rates of water loss by shoots during periods of low rainfall. Consequently, N fertilization enhances the probability of tree death along natural gradients of increasing soil salinity and aridity. The emergence of this pattern across species and biogeographic regions underscores the importance of climatic interactions with the intertidal landscape in determining how coastal ecosystems respond to eutrophication.

At many sites fertilization with N enhanced tree mortality in scrub forests, but not in seaward fringing forests. These differences are probably associated with tidally maintained differences in sediment salinities and their variability. Seaward fringing forests are buffered against negative impacts of nutrient enrichment because regular tidal inundation limits development of hypersalinity in sediment pore water. In contrast, scrub forests are less frequently inundated by tides, and, consequently, are exposed to highly variable soil salinities which can become hypersaline during dry periods. Under these conditions, fertilization with N was associated with increased mortality (Fig S1). There was however variation in the influence of fertilizer among sites indicating factors other than nutrient enrichment may enhance mortality of mangroves during droughts or ameliorate the effects of drought. For example, high mortality in Exmouth in unfertilized and fertilized trees occurred in 2003 during a period of reduced tidal inundation, while low mortality in scrub forests in Belize may attributed to the high frequency and intensity of inundation in scrub forests.

Trees fertilized with P tended to have higher probability of survival than those fertilized with N (Fig S2) and had lower levels of canopy loss than N fertilized trees or unfertilized trees (Fig S3). Positive effects of P fertilizer on the water status of leaves, photosynthetic water use efficiency [18], [31] and hydraulic conductivity of stems [18], [32], [33] indicate that added P improves water relations under high salinity conditions, possibly through enhancing aquaporin function [34]. Thus, the ratio of available N:P at sites enriched in nutrients may be important in determining the resilience of mangrove forests to periodic development of hypersaline soils under conditions of low rainfall and humidity.

Although our study focused on mangroves, our results may be applicable to other ecosystems where anthropogenic nutrient enrichment occurs. For example, McCarthy et al (2006) [35] reported no adverse effects of N fertilisation on forest trees at a site where hot summers were accompanied by high rainfall and high humidity. In contrast, the combination of high N availability followed by drought has been observed to decrease productivity and enhance mortality in tree species of genera as distantly related as *Pinus* [36] and *Eucalyptus* [37]. Similarly, van Herwaarden et al. (1998) [38] found that application of high levels of N fertilizers negatively affected the productivity and yield of wheat when rainfall was low. The occurrence of similar results in such disparate systems is strong evidence of a general pattern in plant responses to high levels of N fertilization with variation in drought.

Fluctuations in rainfall have been strongly implicated in causing changes in ecosystem state from mangrove to salt marsh and cyanobacterial mat in arid regions [39], [40] indicating that die-back of mangroves may be a frequent feature of arid zone intertidal landscapes. Given the strength of the effect of N enrichment on mortality of mangroves, die-back of mangrove forests would be expected to occur in coastal areas that receive large nutrient influxes from anthropogenic sources and are subject to pronounced seasonal or inter-annual variation in rainfall and evaporative demand. Indeed, die-back of mangrove forests has been documented in many regions affected by runoff from agricultural lands. Although these dieback events have often been attributed to a wide range of causes, including herbicides [41] and diseases [42], they have also been associated with periods of low rainfall [43] and high soil salinity [44]. Our study shows that the die-back could also be exacerbated by adverse effects of growth under high nutrient availability on the capacity to survive development of hypersaline soil conditions during droughts.

Our results indicating enhanced instability with coastal eutrophication has far reaching consequences for many aspects of mangrove ecosystem function under contemporary and future climatic conditions. Enhancement of canopy loss and tree mortality would reduce benefits to productivity due to increasing atmospheric [CO_2_], and increase the disparity in productivity between fringe and scrub forest types, with the differences becoming greater along climatic gradients of increasing aridity. This could have greater relative impact on ecosystem function in drier areas where scrub forests account for the majority of mangrove forest cover [40].

Our results show that mangroves exposed to high nutrient availability suffer greater mortality during drought, and that nutrient-induced mortality is greater in sites subject to periods of low rainfall, low humidity and high sediment salinity. However, it would be incorrect to assume that fertilization would have no adverse effects in forests where hot summers are accompanied by high rainfall and humidity. Stimulation of shoot growth relative to root growth by fertilization could make these forests more vulnerable to windthrow and waves associated with intense tropical storms and tsunamis [45]. Thus the benefits of increased mangrove growth in response to coastal eutrophication will be offset by the costs of lower resilience of mangrove forests when exposed to increasing aridity and to other disturbances.

## Materials and Methods

There were 12 study sites where mangrove trees have been fertilized (Table S1). Within each site x forest zone (seaward fringing or landward scrub) replicate trees (6–9 replicates) were fertilized either annually or biannually by inserting 200–300 g of urea (nitrogen, N) or triple superphosphate (Phosphorus, P) into 30 cm deep holes cored on either side of the main stem of the tree. Holes were then sealed with a portion of the extracted sediment core. Control trees were cored but not fertilized. Tree growth was measured as extension of 5 replicate twigs per tree in sun lit positions in the canopy either biennially or annually for a minimum of 2 years. Fertilization continued after growth measurements were completed. All sites have been fertilized for at least 3 years. Climatic summaries for each site were obtained from within country government meteorological services. Porewater was extracted from soil beneath each tree using a suction device and salinity measured with a handheld refractometer [14]. Mortality of trees was recorded at each site during the course of the experiments. Tree mortality (number of trees that were live and dead) over the fertilization treatments was assessed using logistic regression with salinity and rainfall as covariates in the model. Differences in survivorship among treatments were assessed using logits and Chi squared tests. We assessed correlation between % mortality and characteristics of the sites (rainfall and porewater salinity) and the time since fertilization using Kendall's Tau.

At two of the sites (Hinchinbrook Channel and Port Douglas) we observed significant canopy loss during July 2007 which was associated with a prolonged drought in the region (Australian Bureau of Meteorology 2007). We estimated canopy loss as the proportion of twigs that had died, but were still attached to the tree. The effect of nutrient enrichment on the proportion of canopy loss in 2007 at Hinchinbrook Channel and Port Douglas was assessed using analysis of variance (ANOVA) where site was a random effect in the model and fertilization treatment a fixed effect. Data were log transformed prior to analysis.

## Supporting Information

Figure S1 Death of experimental mangrove trees over fertilization treatments and over rainfall and porewater salinity gradients. Mortality increased with decreasing average annual rainfall and increasing porewater salinity. Trees fertilized with Nitrogen (+Nitrogen) show more pronounced sensitivity to rainfall and salinity than trees fertilized with phosphorus (+Phosphorus) and trees that were not fertilized (Control). Analysis by logistic regression indicates significant fertilization treatment x rainfall (P = 0.001) and fertilization treatment x salinity (P = 0.025) effects on mortality.
(8.71 MB TIF)

Figure S2 The probability of survivorship of scrub mangrove trees over fertilization treatments. The probability of survivorship was lower with nitrogen fertilization (+N) than for unfertilized control trees (C) or for phosphorus fertilized (+P) trees (χ2 = 9.73, P = 0.008). Bars are means and standard errors. Data are means and standard errors from 16 scrub mangrove sites. Different letters above the bars indicate significantly different means (P<0.05).
(7.93 MB TIF)

Figure S3 Canopy loss during a drought episode over fertilization treatments at two mangrove sites in north Queensland. Canopy loss was greater in nitrogen fertilized trees (+N) compared to phosphorus fertilized (+P) or unfertilized control trees (C) trees at two sites with varying salinities; Port Douglas (closed bars, 75 ppt) and Hinchinbrook Channel (hatched bars, 60 ppt) in north Queensland. Fertilizer treatment effect was significant (F_2,2_ = 73.06, P = 0.0135). Canopy loss was significantly greater at Port Douglas than Hinchinbrook Channel (F_1,45_ = 6.01, P = 0.018). Data are means and standard errors for 9 trees per treatment at each site.
(8.70 MB TIF)

Table S1 Locations and characteristics of mangrove fertilization experimental sites. Locations and characteristics of mangrove fertilization experimental sites, including climatic variables (average annual temperature and rainfall), tidal range, soil type, species included in the experiment, forest type (seaward fringe or scrub forest), canopy height, number of trees included in each experiment and duration of the experimental observations. Human influences on the site are also indicated and the nutrient that limits growth with the magnitude of the growth enhancement above non-fertilized controls appears in parenthesis.
(0.06 MB DOC)
