# Supplementary material for: Nutrient Enrichment Increases Mortality of Mangroves
Source: PLoS One. 2009 May 19;4(5):e5600. doi: 10.1371/journal.pone.0005600 (PMC2679148; doi:10.1371/journal.pone.0005600)
Supplement: Table S1 — Locations and characteristics of mangrove fertilization experimental sites. Locations and characteristics of mangrove fertilization experimental sites, including climatic variables (average annual temperature and rainfall), tidal range, soil type, species included in the experiment, forest type (seaward fringe or scrub forest), canopy height, number of trees included in each experiment and duration of the experimental observations. Human influences on the site are also indicated and the nutrient that limits growth with the magnitude of the growth enhancement above non-fertilized controls appears in parenthesis. (0.06 MB DOC) [file pone.0005600.s004.doc]

## Table S1

Locations and characteristics of mangrove fertilization experimental sites, including climatic variables (average annual temperature and rainfall), tidal range, soil type, species included in the experiment, forest type (seaward fringe or scrub forest), canopy height, number of trees included in each experiment and duration of the experimental observations. Human influences on the site are also indicated and the nutrient that limits growth with the magnitude of the growth enhancement above non-fertilized controls appears in parenthesis.

| Site | Lat. Long. | Average annual min. and max. air temp in ºC and rainfall (m) | Tidal range (m) | Soil | Species | Forest type | Canopy height | Number of trees | Duration of observations | Human influences identified at the site | Limiting nutrient and proportional growth enhancement above the controls (in parenthesis) |
| --- | --- | --- | --- | --- | --- | --- | --- | --- | --- | --- | --- |
| Bocas del Toro, Panama | 9°21’ N, 82°15’W | 27-30  (~4.0) | 0.7 | Organic-peat | *Rhizophora mangle* L. | Seaward fringe | 3 -5 m | 27 | 6 | Undisturbed, although agriculture within the catchment | N (4.5) |
|  |  |  |  | Organic-peat | *R. mangle* | Scrub | <1.5 m | 27 |  |  | N and P (1.5, 4.6) |
| Port Douglas, Queensland, Australia | 16°30’ S, 145° 27’ E | 20.6-27.9  (2.01) | 2.1 | Mineral-sand | *Avicennia marina* (L.) Forsk.) Vierh. | Scrub | <2 m | 24 | 7 | Undisturbed, although agriculture within the catchment | N (1.8) |
| Twin Cays, Belize | 16°50’ N, 88°06’ W | 25.4-28.8 (~2.5) | 0.5 | Organic-peat | *R. mangle* | Seaward fringe | 5 – 7 m | 27 | 12 | Undisturbed | N (1.5) |
|  |  |  |  | Organic-peat | *R. mangle* | Scrub | < 1.5 m | 27 |  |  | P (31.9) |
|  |  |  |  | Organic-peat | *A. germinans* (L.) Stearn | Scrub | < 2 m | 24 |  |  | P (4.4) |
| Hinchinbrook Channel, Queensland | 18°20’S, 146° 10’ E | 18.8-28.8 (2.12) | 2.3 | Organic-peat | *R. lamarckii* Montr. | Seaward fringe | 5-7m | 27 | 7 | Undisturbed | N and P (4.8, 3.6) |
|  |  |  |  | Mineral - sand | *C. tagal* (Perr.) C.B. Rob. | Scrub | <1.5 m | 27 |  |  | N and P (3.1, 7.3) |
| Cape Cleveland, Queensland, Australia | 19°16’ S, 147° 01’ E | 21.7-27.4  (1.17) | 2.3 | Mineral-sand | *A.marina* | Scrub | < 1.5m | 18 | 7 | Undisturbed | N (3.6) |
|  |  |  |  | Mineral-sand | *C. tagal* | Scrub | < 1.5m | 18 |  | Undisturbed | N (6.7) |
| Giralia, Western Australia | 21°44’ S, 114° 35’ E | 17.7-31.7 (0.26) | 1.6 | Mineral-silt | *A. marina* | Scrub | <1.5 m | 27 | 3 | Undisturbed | N (1.7) |
|  |  |  |  | Mineral-silt | *A. marina* | Seaward fringe | 3 – 5 m | 27 |  |  | N (1.6) |
| Exmouth, Western Australia | 21°58’ S, 113° 57’ E | 17.7-31.7 (0.26) | 1.7 | Mineral-sand | *A. marina* | Scrub | <1.5 | 21 | 4 | Undisturbed | None |
| Brisbane, Queensland | 21°17’ S, 153° 02’ E | 15.7-25.4 (1.19) | 2.7 | Organic peat | *A. marina* | Seaward fringe | 7-10 m | 27 | 3 | Urban and agricultural development in the catchment | None |
|  |  |  |  | Mineral - silt | *A. marina* | Scrub | <2.5 m | 27 |  |  | N (7.5) |
|  |  |  |  | Mineral - silt | *C, tagal* | Scrub | <2 m | 27 |  |  | N (3.7) |
| Fort Pierce, Florida | 27º33'N, 80º20'W | 18.0-27.9 (1.37) | 0.5 | Mineral-silt | *R. mangle* | Seaward fringe | 5-7 m | 27 | 6 | Urban and agricultural development in the catchment | N (4.2) |
|  |  |  |  | Mineral-sand | *A. germinans* | Scrub | < 1.5 m | 27 | 6 |  | N (6.0) |
|  |  |  |  | Mineral-sand | *Laguncularia racemosa* (L.) Gaertn. f. | Scrub | < 1.5 m | 18 | 6 |  | N (4.1) |
| Bateman’s Bay, New South Wales | 32º42’S, 150º12’E | 10.1 – 21.7  (0.9) | 0.9 | Mineral – sand/silt | *A. marina* | Seaward fringe | 3-4 m | 27 | 4 | Forestry in the catchment | N (3.6) |
|  |  |  |  |  | *A. marina* | Scrub | < 1.5 m | 27 |  |  | N (2.5) |
| Whangapoua, New Zealand | 36°45’S, 175°30’E | 11.4-19.6 (2.0) | 1.6 | Mineral-sand | *A. marina* | Seaward fringe | 2-3 m | 24 | 3 | Forestry in the catchment | N (1.7) |
|  |  |  |  | Mineral-sand | *A. marina* | Scrub | <1.5 | 24 |  |  | N (2.1) |
| Waikopua, New Zealand | 36° 55’ S  174° 30’E | 11.3-18.9 (2.5) | 2.4 | Mineral-silt/mud | *A. marina* | Seaward fringe | 2-3 m | 24 | 3 | Urban and agricultural development in the catchment | N (1.1) |
|  |  |  |  | Mineral-silt/mud | *A. marina* | Scrub | < 1.5 m | 24 |  |  | N (10.2) |
